# Effect of Autonomous Vehicles on Fatigue Life of Orthotropic Steel Decks

**DOI:** 10.3390/s22239353

**Published:** 2022-12-01

**Authors:** Shengquan Zou, Dayong Han, Wei Wang, Ran Cao

**Affiliations:** 1 College of Civil Engineering, Hunan University, Changsha 410012, China; 2 Powerchina Road Bridge Group Co., Ltd., Zhongshan 528405, China; 3 Key Laboratory of Damage Diagnosis for Engineering Structures of Hunan Province, Hunan University, Changsha 410012, China

**Keywords:** autonomous vehicle, transverse distribution of vehicles, orthotropic steel deck (OSD), lightweight composite OSD (LWCD), fatigue life

## Abstract

The fatigue life of orthotropic steel decks (OSDs) is significantly affected by vehicle loads, and the local stress response of OSDs is sensitive to the transverse position of vehicle loads. However, the presence of autonomous vehicles is likely to change the transverse distribution of vehicles within the lane, thereby affecting vehicle-induced fatigue damage to OSDs. Therefore, it is necessary to evaluate the potential effect of autonomous vehicles on the fatigue life of OSDs so that appropriate strategies can be implemented to control the transverse positions of autonomous vehicles passing the bridge deck. To this end, fatigue damages of several typical fatigue details in a conventional OSD (COSD) and a lightweight composite OSD (LWCD) induced by vehicle loads were calculated based on finite element analysis, and their fatigue lives were evaluated based on Miner’s Rule, in which different transverse distribution patterns of autonomous vehicles and their proportions in the mixed traffic flow were considered. The results indicate that fatigue lives of both the COSD and the LWCD can be negatively affected by autonomous vehicles traveling across the bridge without any constraints on the transverse distribution, especially when their proportion in the mixed traffic flow exceeds 30%. Compared to the scenario without autonomous vehicles, the fatigue damage of most fatigue details in OSDs may increase by 51% to 210% in the most unfavorable case due to the presence of autonomous vehicles. Nevertheless, it is feasible to extend the fatigue life of OSDs by optimizing the transverse distribution of autonomous vehicles. Specifically, the fatigue life of most fatigue details in the COSD could be extended by more than 86% in the most favorable case when a bimodal Gaussian distribution is adopted as the transverse distribution pattern of autonomous vehicles. Moreover, both the negative and positive effects of autonomous vehicles on the fatigue life of the COSD are more significant than those of the LWCD in most cases. The results can provide references for the maintenance of OSDs under the action of autonomous vehicles.

## 1. Introduction

Orthotropic steel decks (OSDs) consist of flat steel plates stiffened by longitudinal ribs and transverse crossbeams, which have different stiffness characteristics in two perpendicular directions. Owing to its advantages of lightweight, high strength, convenient construction, and low life-cycle cost, the OSD has been extensively used in medium and large-span bridges all over the world [1,2]. However, OSDs are prone to fatigue cracking due to the complicated details and welds, which may affect the operation of bridges and even endanger the safety of bridges [3,4,5]. Furthermore, this phenomenon has become increasingly common and severe in conventional OSDs (COSDs) with the increase in vehicle loads and traffic volume, resulting in great economic losses and serious safety risks [6]. In recent years, various efforts have been made to improve the fatigue performance of OSDs. Thereinto, the lightweight composite OSD (LWCD), which is mainly composed of an OSD and an ultrahigh performance concrete (UHPC) layer, has been demonstrated to have a good performance in extending the fatigue life of the OSD [7] and is expected to be vigorously promoted and widely applied [8].

As a matter of fact, the fatigue life of OSDs is closely related not only to the properties of materials and structures but also to the vehicle loads acting directly on the bridge deck [3]. It has been found that the stress response of fatigue details in OSDs is quite sensitive to the position of vehicle loads [9] and that different transverse distributions of vehicle loads may cause a difference of more than 14% in the equivalent stress range of these fatigue details [10]. Similarly, the stress response of steel bridge deck in the LWCD is also significantly affected by the transverse position of vehicle loads though the local stiffness of bridge decks in the LWCD has been greatly improved due to the UHPC layer [11]. Furthermore, some structural details in the LWCD are still at risk of fatigue cracking [12,13]. Therefore, the influence of the transverse distribution of vehicles on the fatigue life of both COSDs and LWCDs cannot be ignored.

It has been confirmed that the transverse positions of human-driven vehicles within a lane can be assumed to follow a normal distribution centered on the lane centerline, which has been adopted by numerous researchers and bridge design specifications [14]. However, the driving behavior of autonomous vehicles may be different from that of human drivers since self-driving technology can break through the limitation of human drivers [15]. As a result, the transverse distribution pattern of vehicles is likely to be changed due to the presence of autonomous vehicles. Currently, the trajectory-oriented method is mainly adopted in the self-driving control system to ensure driving safety and thus autonomous vehicles are generally controlled to travel along the lane centerline as much as possible [16,17], which will affect the stress behavior of fatigue details in OSDs and thus affect their fatigue lives. In fact, many vehicles sold in recent years have been equipped with self-driving functions, and more-automated vehicles are continuously tested on public roads [18]. With the massive investment in self-driving technology, fully autonomous vehicles are expected to become a reality in the 21st century [19]. At present, apart from driving safety, many studies have also focused on the effect of autonomous vehicles on traffic efficiency, energy saving, environmental protection, and so on [20]. However, the effect of autonomous vehicles on the performance of transport infrastructures has received limited attention [21]. Therefore, there is an urgent need to reveal the potential effect of autonomous vehicles on the fatigue life of OSDs.

The trajectory tracking of autonomous vehicles is increasingly reliable and controllable as technology improves [22]. From a technical perspective, the transverse distribution of autonomous vehicles can be controlled to follow a specified distribution pattern [16]. Considering that the stress of almost all fatigue details in OSDs fluctuates greatly when the transverse position of vehicle loads changes [9], it is theoretically feasible to reduce the fatigue damage of fatigue details in OSDs by optimizing the transverse distribution of autonomous vehicles. In this way, the negative effect of autonomous vehicles on the fatigue life of OSDs may be turned into a positive one, which may provide a new way to extend the fatigue life of OSDs. It should be noted that the vehicles investigated in this paper only refer to trucks since the fatigue life of OSDs is mainly affected by truck traffic and self-driving technology has also been widely applied in autonomous trucks [16].

This paper aims to evaluate the potential effect of autonomous vehicles on the fatigue life of OSDs and provide references for the maintenance of OSDs under the action of autonomous vehicles. Finite element models (FEMs) of a COSD and an LWCD were built according to the girder segment of OSDs of a cable-stayed bridge. The stress responses of several typical fatigue details in these two OSDs were obtained as the vehicle loads were applied at different transverse positions. Based on Miner’s Rule, the equivalent fatigue damages of these fatigue details were calculated and the effects of autonomous vehicles on the fatigue life of both the COSD and the LWCD were evaluated, in which different transverse distribution patterns of autonomous vehicles and their proportions in the mixed traffic flow were considered. Additionally, the feasibility of extending the fatigue life of OSDs by optimizing the transverse distribution of autonomous vehicles was evaluated based on two proposed transverse distribution patterns of vehicles. Finally, the negative effect of autonomous vehicles on the fatigue life of OSDs was evaluated quantitatively if their transverse distribution is unconstrained and a new way to extend the fatigue life of OSDs based on the optimization of the transverse distribution of autonomous vehicles was provided and verified.

## 2. Finite Element Analysis

In order to evaluate the potential effect of autonomous vehicles on the fatigue life of OSDs, two representative OSD systems and several typical fatigue details in these OSDs were considered in the case study, which is described in this section. Furthermore, the details on the finite element model and fatigue load adopted in the study were introduced.

### 2.1. OSD Systems

**Figure 1:**
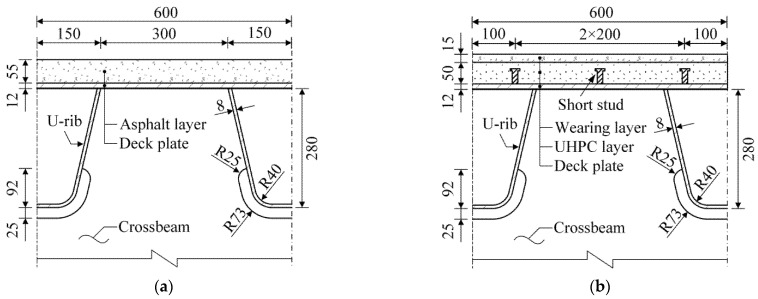
Details and dimensions of OSDs (Unit: mm): (**a**) COSD; (**b**) LWCD.

COSDs have been applied to a lot of bridges around the world, which will remain in service for many years. Meanwhile, the LWCD is expected to be widely used in the construction of new bridges and the renovation of existing ones due to its excellent fatigue performance [8]. In this paper, these two OSD systems were therefore considered to evaluate the influence of autonomous vehicles on the fatigue life of OSDs. Details and dimensions of these OSD systems were obtained from a cable-stayed bridge, as illustrated in Figure 1. In the COSD, an asphalt layer is directly paved on the deck plate of an OSD, while in the LWCD, a UHPC layer is laid between the wearing layer and the OSD, which is connected to the deck plate by short studs [23]. The thickness of deck plates in the COSDs built in recent years commonly ranges from 14 to 24 mm, while it can be reduced to 12 mm in the LWCD as the UHPC layer considerably improves the local stiffness of the bridge decks [24]. In fact, the 12-mm-thick deck plate is also used in the OSDs built before 2000, which are still in service and are more susceptible to fatigue cracking, such as the Humen Bridge in China. In this paper, the thicknesses of deck plates in those two OSDs were intendedly set as 12 mm for comparison.

### 2.2. Fatigue Details

**Figure 2:**
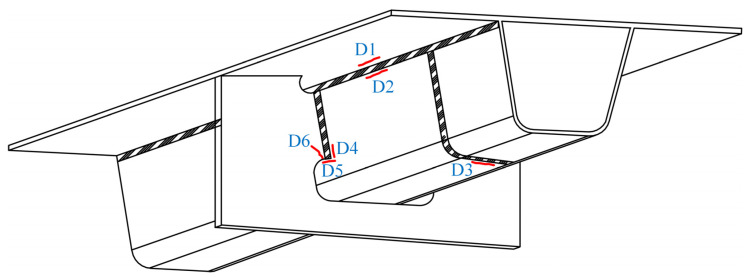
Fatigue details in OSDs.

Many fatigue details in OSDs are prone to fatigue cracking due to the crisscrossing components and dense welds. More than 17 types of fatigue details have been found in steel bridge decks [6]. In this paper, six typical fatigue details identified in OSDs were considered, as shown in Figure 2, where D1 is the welded joint of rib-to-deck (RD) in deck plates, D2 is the welded joint of RD in ribs, D3 is the welded joint in the splice of ribs, D4 is the welded joint of rib-to-crossbeam (RC) in ribs, D5 is the end of the welded joint of RC in ribs, and D6 is the welded joint of RC in crossbeams. Note that the fatigue details D1, D2, and D3 in the LWCD were not investigated in this paper since their stress ranges induced by vehicle loads are below the corresponding cut-off limit, indicating that they have no risk of fatigue cracking under normal conditions [11,25].

### 2.3. Finite Element Models

**Figure 3:**
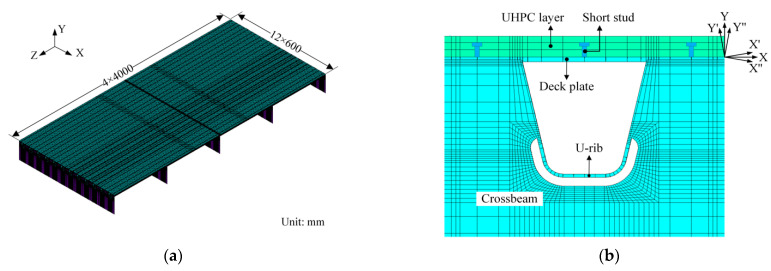
Local FEM of LWCD: (**a**) Full view of FEM; (**b**) Detail view of interest.

Generally, the fatigue damage of fatigue details in OSDs is mainly induced by vehicles crossing the bridge decks and it has been found that the affected area of vehicle loads is rather limited [9]. Therefore, the local FEM of OSDs is sufficient to accurately obtain the stress response of fatigue details required for fatigue evaluation [26]. According to the design of these OSDs illustrated in Figure 1, local FEMs of the COSD and the LWCD were established based on the general software of ANSYS. Note that the role of the asphalt layer in the COSD was replaced by the load dispersion instead of establishing elements in the FEM, which is an accepted practice assuming that wheel loads are dispersed through the asphalt overlay with an angle of 45 degrees [2]. Meanwhile, the role of the wearing layer was ignored in the local FEM of the LWCD as it is too thin. In this way, the local FEM of the COSD differs from that of the LWCD only in the UHPC layer and short studs. The local FEM of the LWCD is shown in Figure 3 for illustration, which has a length of 16 m and a width of 7.2 m and includes five crossbeams and twelve U ribs. The key parameters of the present FEMs are listed in Table 1.

It has been confirmed that the accuracy of the stress response of OSDs obtained from the local FEM can be guaranteed when the mesh size of the elements of interest does not exceed 5 mm [27]. Coarser meshes can be accepted for the higher-order element, but the element size is usually no more than 10 × 10 mm or *t* × *t* (*t* is the plate thickness) [28,29]. To balance the calculation efficiency and accuracy, fine mesh with a length of 0.5 × *t* was performed for the elements near the fatigue details under consideration and the minimum element size was 4 × 4 mm, while the mesh size of the elements away from the fatigue details increases gradually. Overall, the local FEM of the LWCD contains a total of 400,596 elements and 449,784 nodes.

The global coordinate system in the present FEMs was defined as illustrated in Figure 3a. The X’-axis, Y’-axis, X’’-axis, and Y’’-axis in the local coordinate system, as shown in Figure 3b, were defined according to the angles of the webs on both sides of the U-rib. The translational degrees of freedom (DOFs) in the *Z*-axis and the rotational DOFs around the *X*-axis and *Y*-axis of all nodes at both ends of the present FEMs, except those of the end crossbeams, were constrained to model the role of continuous bridge decks. The translational DOFs in the *X*-axis and the rotational DOFs around the *Y*-axis and *Z*-axis of all nodes on the sides of the present FEMs were constrained to model the role of adjacent bridge decks. The translational DOFs in the *Y*-axis of the nodes at the bottom of all crossbeams were constrained to simulate the support of crossbeams. According to the Saint Venant principle, the stress response of fatigue details far away from the boundary of FEM would be hardly affected by those boundary constraints [30]. In the local FEM of the LWCD, the upper and bottom nodes of short studs were coupled with the corresponding nodes in the UHPC layer and the deck plate, respectively, ignoring the effect of nonlinear bonding between the UHPC layer and the short studs. The translational DOFs in the *Y*-axis of the nodes at the contact surface between the UHPC layer and the deck plate were coupled, ignoring the effect of bond-slip and push-out between them. Furthermore, the nonlinearity of materials was not considered in this paper as the stress of components in OSDs induced by vehicle loads generally remains linear-elastic [7]. It should be noted that these finite element modeling strategies used in this paper have been proven to be acceptable and effective to analyze the stress response of OSDs [24,31].

### 2.4. Fatigue Load

**Figure 4:**
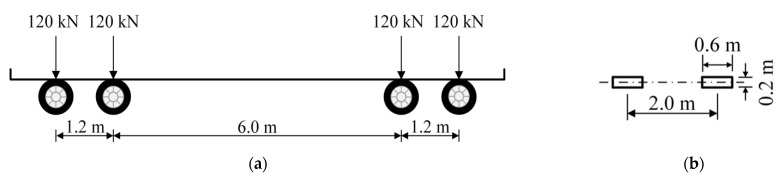
Fatigue load model: (**a**) Axle arrangement; (**b**) Standard axle.

The fatigue load used in this paper was adopted from the Chinese specification for the design of highway steel bridges [32], namely, Fatigue Load Model (FLM) 3. As plotted in Figure 4, FLM 3 is a standard fatigue truck with a total weight of 480 kN evenly distributed on four standard axles. Furthermore, it was assumed that the roughness of the pavements in both the COSD and the LWCD is of good quality, thereby the dynamic amplification factor was set to be 1.2 [33].

To evaluate the effect of autonomous vehicles on the fatigue life of OSDs, it is necessary to obtain the stress history of fatigue details in OSDs as vehicles pass the bridge from different transverse positions. For this purpose, the influence area of the stress of each fatigue detail under consideration was obtained when the fatigue truck of FLM 3 was applied on the bridge deck with a longitudinal (*Z*-axis) loading step of 0.10 m and a transversal (*X*-axis) loading step of 0.05 m.

## 3. Transverse Distribution of Vehicles

Unlike a human-driven vehicle, the transverse position of autonomous vehicles can be program-controlled. Therefore, the transverse distribution of autonomous vehicles in a lane may be significantly different from that of human-driven vehicles, resulting in different effects on the fatigue life of OSDs. In this section, the transverse distributions of human-driven vehicles and autonomous vehicles were first determined based on mathematical models, respectively. Then, the transverse distribution of the mixed traffic flow consisting of autonomous vehicles and human-driven vehicles was discussed.

### 3.1. Human-Driven Vehicles

The accurate transverse position of a human-driven vehicle is difficult to predict, which depends on the driver’s driving preference and operational stability [34]. Furthermore, the transverse distribution pattern of human-driven vehicles is certainly affected by road class and vehicle type [35]. Nevertheless, it is available to count the transverse positions of all vehicles within a lane, and then their transverse distribution pattern can be obtained through data fitting. It has been indicated that the transverse positions of human-driven vehicles can be assumed to follow a normal distribution [36], which has also been confirmed by Kim [37] based on the measured data. Referring to the data of heavy trucks measured from a freeway [38], it was assumed that the transverse positions of human-driven vehicles follow a normal distribution with a standard deviation of 30 cm and are centered on the lane centerline. Furthermore, all vehicles were assumed to travel straight across the bridge deck and thus vehicles changing lanes were ignored.

**Figure 5:**
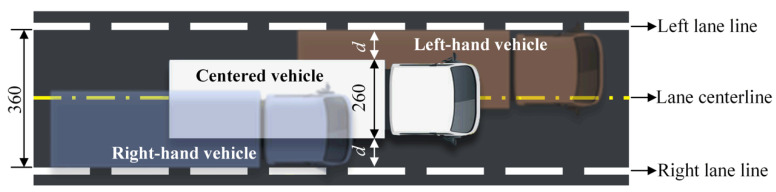
Transverse positions of vehicles within a lane (Unit: cm).

The clear width of a lane is 3.6 m and the width of the contour of FLM 3 is 2.6 m, as illustrated in Figure 5. When the standard fatigue truck travels within the lane, its maximum transverse offset is (360 − 260)/2 = 50 cm. Considering that the width of real trucks usually does not exceed 2.5 m and that human-driven vehicles may cross the lane line, the maximum offset of human-driven vehicles from the lane centerline was assumed to be 60 cm in this paper. For convenience, the vehicle center was taken as the reference point of the vehicle position and the lane centerline was set as the zero point of the transverse coordinate. In this way, all human-driven vehicles can be regarded to be distributed within a transverse range of (−60 cm, 60 cm). Then, the probability density function (PDF) of the transverse distribution of human-driven vehicles can be modified as [38]:

(1) $$F_{h}\left(x\right)=\left\{ \begin{array}{l} \frac{f_{h}\left(x\right)}{\int_{-60}^{60}f_{h}\left(x\right)\cdot dx},-60\leq x\leq 60\\ 0,x<-60\;\mathrm{or}\;x>60 \end{array}\right.$$

where *x* is the transverse offset of the vehicle from the lane centerline; and *f*_h_(*x*) is the original PDF of the transverse distribution of human-driven vehicles, *f*_h_(*x*)~*N*(*μ*_h_, $\sigma_{h}^{2}$), in which *μ*_h_ and *σ*_h_ are the mean and standard deviation of *f*_h_(*x*), taking a value of 0 and 30 cm, respectively.

**Figure 6:**
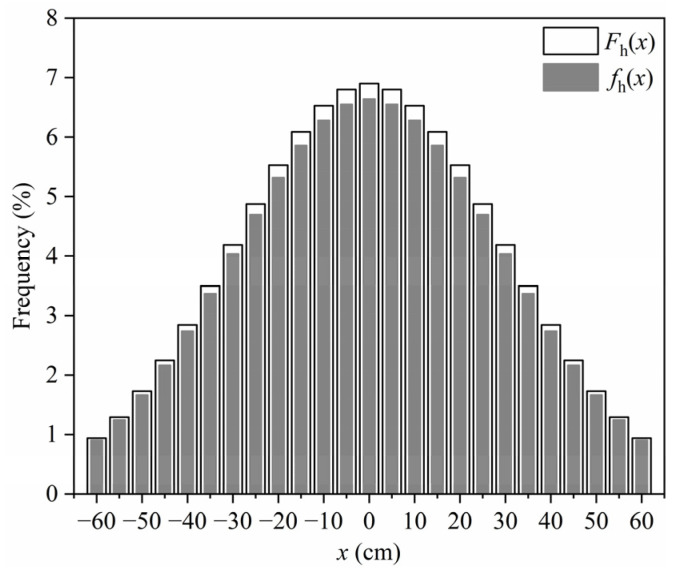
Transverse distribution frequency of human-driven vehicles.

Based on Equation (1), the frequency of human-driven vehicles traveling along different transverse positions within a lane is illustrated in Figure 6, and the corresponding frequency calculated based on the original PDF is also included for comparison. Note that the transverse range of (−60 cm, 60 cm) was divided into 25 stripes with a segment distance of 5 cm to simplify calculations. It can be found from Figure 6 that the transverse distribution frequency of human-driven vehicles calculated based on the modified PDF is very close to that calculated based on the original PDF, indicating that it is acceptable to use the modified PDF to express the transverse distribution of human-driven vehicles.

### 3.2. Autonomous Vehicles

At present, the studies on transverse control technology of autonomous vehicles mainly focus on improving driving stability and safety through deep learning of human driving behavior. If unconstrained, the transverse distribution of autonomous vehicles would also be approximated with a normal distribution [17]. Therefore, it was assumed that the transverse positions of autonomous vehicles still follow a normal distribution centered on the lane centerline in this paper. Unlike human-driven vehicles, autonomous vehicles can avoid the poor driving behavior of human drivers. Hence, the case of autonomous vehicles crossing the lane line can be eliminated so that all autonomous vehicles were regarded to be distributed within a transverse range of (−50 cm, 50 cm) in this paper. Similarly, the PDF of the transverse distribution of autonomous vehicles can be expressed as

(2) $$F_{a}\left(x\right)=\left\{ \begin{array}{l} \frac{f_{a}\left(x\right)}{\int_{-50}^{50}f_{a}\left(x\right)\cdot dx},-50\leq x\leq 50\\ 0,x<-50\;\mathrm{or}\;x>50 \end{array}\right.$$

where *f*_a_(*x*) is the original PDF of the transverse distribution of autonomous vehicles, *f*_a_(*x*)~*N*(*μ*_a_, $\sigma_{a}^{2}$), in which *μ*_a_ and *σ*_a_ are the mean and standard deviation of *f*_a_(*x*), respectively, and are affected by the robustness of the vehicle control system and the effect of vehicle wander.

**Figure 7:**
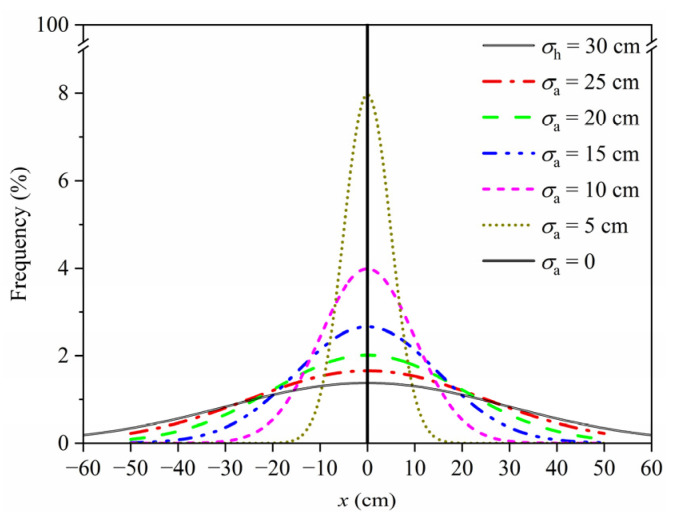
PDF of transverse distribution of vehicles.

The current self-driving technology has been able to ensure that autonomous vehicles travel along the lane centerline with a deviation of less than 10 cm [38]. For the purpose of this study, *μ*_a_ was taken as 0 and five values for *σ*_a_ were considered, namely, 25 cm, 20 cm, 15 cm, 10 cm, 5 cm, and 0, wherein *σ*_a_ = 0 indicates that autonomous vehicles can maintain excellent stability to track the lane centerline without any offset. According to Equation (2), the modified PDFs of the transverse distribution of autonomous vehicles considering different standard deviations are illustrated in Figure 7, in which the modified PDF of the transverse distribution of human-driven vehicles (*σ*_h_ = 30 cm) was also plotted for comparison. As can be seen from Figure 7, the frequency of autonomous vehicles traveling along different transverse positions may be significantly different from that of human-driven vehicles.

### 3.3. Mixed Traffic Flow

At present, some vehicles equipped with self-driving functions are already on the road. It can be predicted that more human-driven vehicles will be gradually replaced by autonomous vehicles and that the future traffic flow will consist of autonomous vehicles and human-driven vehicles, termed mixed traffic flow in this paper. Therefore, the proportion of autonomous vehicles in the mixed traffic flow was also considered in this study. Combining Equations (1) and (2), the PDF of the transverse distribution of the mixed traffic flow can be calculated as

(3) $$F_{m}(x)=(1-p)\cdot F_{h}\left(x\right)+p\cdot F_{a}(x)$$

where *p* is the proportion of autonomous vehicles in the mixed traffic flow; and *F*_h_(*x*) and *F*_a_(*x*) are the PDF of the transverse distribution of human-driven vehicles and autonomous vehicles, referring to Equations (1) and (2), respectively.

**Figure 8:**
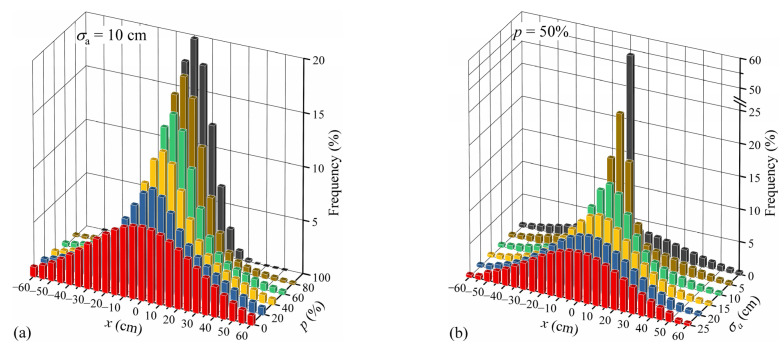
Transverse distribution frequency of the mixed traffic flow: (**a**) Case of *σ*_a_ = 10 cm; (**b**) Case of *p* = 50%.

Similar to the stripe division shown in Figure 6, the transverse distribution frequencies of the mixed traffic flow under two scenarios, namely *σ*_a_ = 10 cm and *p* = 50%, are illustrated in Figure 8 for examples. It can be found that the transverse distribution frequency of the mixed traffic flow is significantly affected by *p* or *σ*_a_. Under the scenario with *σ*_a_ = 10 cm, as shown in Figure 8a, the probability of vehicles traveling along the lane centerline when *p* = 100% is about 2.9 times that when *p* = 0. Under the scenario with *p* = 50%, as shown in Figure 8b, the probability of vehicles traveling along the lane centerline when *σ*_a_ = 0 is even about 7 times that when *σ*_a_ = 25 cm. It demonstrates that vehicles would be excessively concentrated on the lane centerline if the transverse distribution of autonomous vehicles is unconstrained.

## 4. Fatigue Evaluation of OSDs Considering Autonomous Vehicles

In this section, the method for obtaining the stresses of fatigue details in the COSD and the LWCD under the action of vehicle loads was described and the process to calculate the equivalent fatigue damage of fatigue details was introduced based on Miner’s rule. Finally, the effect of autonomous vehicles on the fatigue damage of each fatigue detail of interest was evaluated under different scenarios.

### 4.1. Stress Calculation of Fatigue Details

The fatigue performance of steel bridges is mainly evaluated based on the *S*–*N* curve, in which the stress of fatigue details of interest is usually determined by the nominal stress method and the hot spot stress method [2]. In general, the nominal stress can be directly obtained from finite element analysis, while the hot spot stress can be calculated based on the stresses of reference points. Surface stress extrapolation is a commonly used method to determine hot spot stress. The extrapolation path, including the linear extrapolation based on two reference points and the quadratic extrapolation based on three reference points, depends on the type of hot spots and the mesh size of elements. According to the International Institute of Welding [29], the hot spot is classified into two categories: (1) weld toe on the plate surface, classified as Type A; (2) weld toe at the plate edge, classified as Type B. Detailed information of fatigue details under consideration is listed in Table 2. It should be noted that the stress of the fatigue detail D3 was directly determined based on the nominal stress method as it is located at a continuous joint.

**Figure 9:**
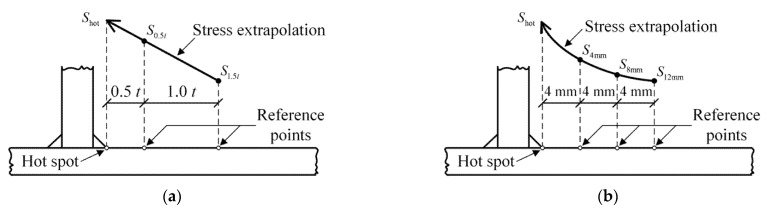
Surface stress extrapolation of hot spots: (**a**) Linear extrapolation; (**b**) Quadratic extrapolation.

The linear extrapolation path is illustrated in Figure 9a and the hot spot stress (*S*_hot_) can be calculated as [29]:

(4) $$S_{\mathrm{hot}}=1.5\times S_{0.5t}-0.5\times S_{1.5t}$$

where *t* is the thickness of the plate on which the weld toe is located; *S*_0.5*t*_ and *S*_1.5*t*_ are the stresses at reference nodes that are 0.5 × *t* and 1.5 × *t* away from the node corresponding to the fatigue detail of interest, respectively.

The quadratic extrapolation path is illustrated in Figure 9b and the hot spot stress can be calculated as [29]:

(5) $$S_{\mathrm{hot}}=3\times \left(S_{4\mathrm{mm}}-S_{8\mathrm{mm}}\right)+S_{12\mathrm{mm}}$$

where *S*_4mm_, *S*_8mm_, and *S*_12mm_ are the stresses at reference nodes that are 4, 8, and 12 mm away from the node corresponding to the fatigue detail of interest, respectively.

### 4.2. Equivalent Fatigue Damage

The *S–N* curve in Eurocode 3 [39] was adopted for the fatigue evaluation in the present study, as expressed below:

(6) $$\left\{ \begin{array}{l} N_{i}\cdot {\left(\Delta S_{i}\right)}^{3}=K_{C},\;\Delta S_{i}>\Delta \sigma_{D}\\ N_{j}\cdot {\left(\Delta S_{j}\right)}^{5}=K_{D},\;\Delta \sigma_{D}\geq \Delta S_{j}>\Delta \sigma_{L} \end{array}\right.$$

where Δ*S_i_* and Δ*S_j_* are the *i*-th high-stress range and the *j*-th low-stress range, respectively; *N_i_* and *N_j_* are the numbers of stress cycles corresponding to the stress ranges of Δ*S_i_* and Δ*S_j_*, respectively, at which fatigue failure occurs; Δ*σ*_D_ and Δ*σ*_L_ are the constant amplitude fatigue limit and the cut-off limit determined based on the detail category (Δ*σ*_C_) of fatigue details, taking a value of Δ*σ*_D_ = 0.737 × Δ*σ*_C_ and Δ*σ*_L_ = 0.549 × Δ*σ*_D_, respectively; *K_C_* and *K_D_* are the fatigue strength constant corresponding to the high-stress range and low-stress range, calculated as *K_C_* = 2 × 10^6^ × (Δ*σ*_C_)^3^ and *K_D_* = 5 × 10^6^ × (Δ*σ*_D_)^5^, respectively.

According to Miner’s rule [40], the cumulative fatigue damage of the fatigue details in OSDs can be evaluated as follows:

(7) $$D=\sum \frac{n_{i}\cdot {\left(\Delta S_{i}\right)}^{3}}{K_{C}}+\sum \frac{n_{j}\cdot {\left(\Delta S_{j}\right)}^{5}}{K_{D}}$$

where *n_i_* and *n_j_* are the numbers of actual stress cycles experienced by the fatigue detail of interest corresponding to the stress ranges of Δ*S_i_* and Δ*S_j_*, respectively.

Previous research by the authors [25] has revealed that the stress reversals experienced by fatigue details in OSDs, which are caused by trucks crossing the bridge from different transverse positions, cannot be ignored when evaluating the fatigue life of OSDs. To this end, the following procedures were performed to calculate the fatigue damage of the fatigue details concerned in this paper. First, the transverse position of each vehicle in the mixed traffic flow consisting of *N* vehicles was randomly generated based on Equation (3), denoted as $V=\left\{v_{1},v_{2},v_{3}\ldots v_{N}\right\}$. Secondly, the stress history of fatigue details of interest induced by a standard fatigue truck, FLM 3, traveling across the bridge separately from each transverse position corresponding to ***V*** was extracted based on finite element analysis, denoted as $S_{v_{k}}$ (*v_k_* represents the transverse position of the *k*-th vehicle). Then, the stress history of fatigue details under the action of each vehicle in the mixed traffic flow was concatenated according to the sequence of ***V***, and the total stress history under the action of the mixed traffic flow was obtained, denoted as $S_{N}=\left\{S_{v_{1}},S_{v_{2}},S_{v_{3}}\ldots S_{v_{N}}\right\}$. Finally, the equivalent fatigue damage of fatigue details under the action of a single standard fatigue truck can be calculated based on Equation (7) and expressed as Equation (8), in which the effect of stress reversals and the transverse distribution of vehicles were considered.

(8) $$D_{e}=\frac{1}{N}\cdot \left[\sum \frac{n_{N,i}\cdot {\left(\Delta S_{N,i}\right)}^{3}}{K_{C}}+\sum \frac{n_{N,j}\cdot {\left(\Delta S_{N,j}\right)}^{5}}{K_{D}}\right]$$

where Δ*S_N,i_* and Δ*S_N,j_* are the *i*-th high-stress range and the *j*-th low-stress range experienced by the fatigue detail of interest, respectively, which are induced by the mixed traffic flow and are extracted from ***S_N_*** using rain-flow counting method; *n_N,i_* and *n_N,j_* are the total numbers of stress cycles corresponding to the stress ranges of Δ*S_N,i_* and Δ*S_N,j_*, respectively; *N* is the number of vehicles in the mixed traffic flow, taken as 1 × 10^4^ [25].

### 4.3. Fatigue Damage of Fatigue Details Considering Autonomous Vehicles

**Figure 10:**
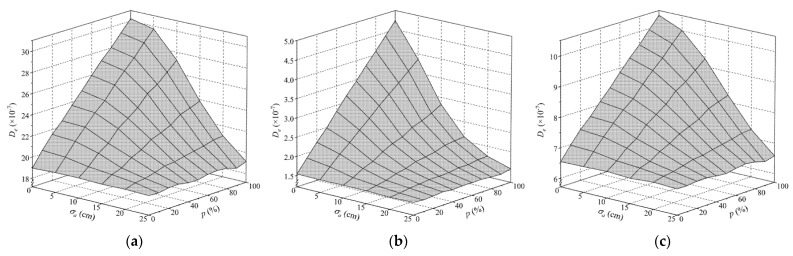

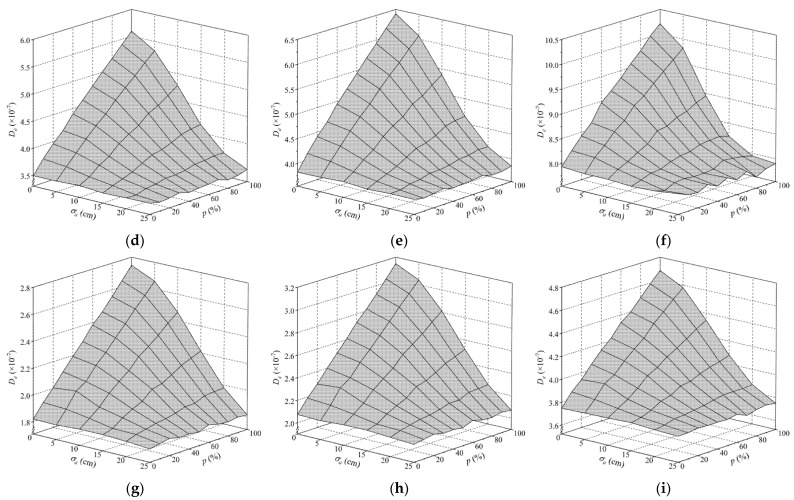
Equivalent fatigue damage of fatigue details induced by a single standard fatigue truck: (**a**) D1 in COSD; (**b**) D2 in COSD; (**c**) D3 in COSD; (**d**) D4 in COSD; (**e**) D5 in COSD; (**f**) D6 in COSD; (**g**) D4 in LWCD; (**h**) D5 in LWCD; (**i**) D6 in LWCD.

The equivalent fatigue damage (*D_e_*) of each fatigue detail concerned in the COSD and the LWCD was calculated based on Equation (8). Note that the most unfavorable transverse position of vehicle loads was assumed to be located at the lane centerline as the future traffic pattern on the bridge is uncertain [2]. Different standard deviations of the transverse distribution of autonomous vehicles, expressed with *σ*_a_ in Equation (2), and different proportions of autonomous vehicles in the mixed traffic flow, expressed with *p* in Equation (3), were considered. The results of *D_e_* of each fatigue detail under different scenarios are shown in Figure 10.

As plotted in Figure 10, *D_e_* of each fatigue detail in OSDs remains constant when there are no autonomous vehicles in the traffic flow, namely in the case of *p* = 0. Conversely, *D_e_* of these fatigue details significantly increases with the decrease in *σ*_a_ or the increase in *p* due to the presence of autonomous vehicles in the traffic flow, namely in the case of *p* > 0. Specifically, *D_e_* of most fatigue details under consideration would increase significantly if *σ*_a_ ≤ 15 cm and *p* ≥ 30%. Compared to the case of *p* = 0, *D_e_* of most fatigue details will increase by 51% to 210% in the extreme case of *σ*_a_ = 0 and *p* = 100%. The results indicate that the fatigue life of OSDs can be significantly affected by autonomous vehicles, especially when more autonomous vehicles are concentrated on the lane centerline. In addition, *D_e_* of different fatigue details in OSDs changes to different extents under the same scenario. In particular, *D_e_* of the fatigue detail D6 in COSD is 7.91 × 10^−7^ when *p* = 0, while it reaches 9.82 × 10^−7^ with an increase of 24% when *σ*_a_ = 5 cm and *p* = 100%. However, *D_e_* of the fatigue detail D2 in COSD increases from 1.53 × 10^−7^ to 3.86 × 10^−7^ under the same case, and the increase in *D_e_*. reaches 152%. Comparing the results of fatigue details D4, D5, and D6 in the COSD and the LWCD, it can be found that *D_e_* of each fatigue detail in the LWCD is much less than that of the COSD, which demonstrates that the LWCD has better fatigue performance than the COSD. Nevertheless, the fatigue damage of fatigue details in the LWCD is still significantly affected by autonomous vehicles, which is just slighter than that of the COSD under the same scenario.

## 5. Optimizing the Transverse Distribution of Autonomous Vehicles

The trajectory of autonomous vehicles is program-controlled based on the real driving environment. It is technically feasible to control the transverse distribution pattern of autonomous vehicles as required. Theoretically, the fatigue damage of fatigue details can be minimized, and thus the fatigue life of OSDs will be maximally extended if all autonomous vehicles are programmed to travel along the bridge deck from the most favorable path without any wandering. However, the deterioration of the pavement would be accelerated to some extent [21], which will in turn affect the fatigue reliability of OSDs [41]. Therefore, the transverse distribution of autonomous vehicles should be optimized to reduce the vehicle-induced fatigue damage of fatigue details while avoiding excessive concentration of wheel tracks. To this end, two distributions, denoted Pattern 1 and Pattern 2, were proposed to optimize the transverse distribution of autonomous vehicles in this paper.

**Figure 11:**
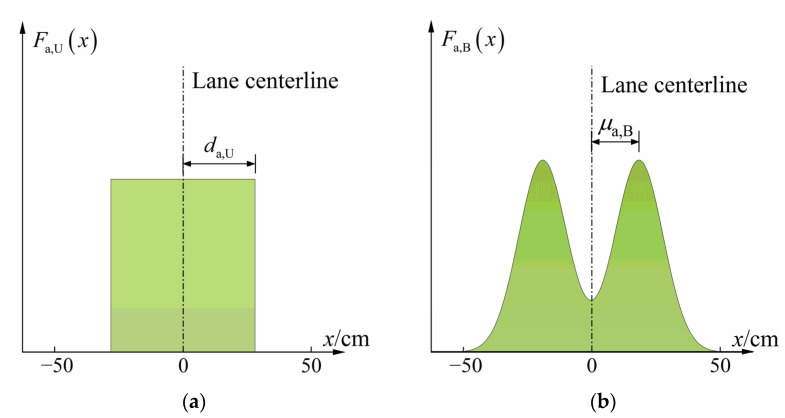
PDF of transverse distribution of autonomous vehicles: (**a**) Pattern 1; (**b**) Pattern 2.

As shown in Figure 11a, transverse positions of autonomous vehicles under the scenario of Pattern 1 follow a uniform distribution centered on the lane centerline, and the PDF can be expressed as

(9) $$F_{a,U}\left(x\right)=\left\{ \begin{array}{l} \frac{1}{2d_{a,U}},-d_{a,U}\leq x\leq d_{a,U}\\ 0,x<-d_{a,U}\;\mathrm{or}\;x>d_{a,U} \end{array}\right.$$

where *d*_a,U_ is the maximum offset of the autonomous vehicle from the lane centerline, taken as 10 to 50 cm.

As shown in Figure 11b, transverse positions of autonomous vehicles under the scenario of Pattern 2 follow a bimodal Gaussian distribution with the lane centerline as the symmetric center, and the PDF can be expressed as

(10) $$F_{a,B}\left(x\right)=\left\{ \begin{array}{l} \frac{f_{a,B}\left(x\right)}{\int_{-50}^{50}f_{a,B}\left(x\right)\cdot dx},-50\leq x\leq 50\\ 0,x<-50\;\mathrm{or}\;x>50 \end{array}\right.$$

where *f*_a,B_(*x*) is the original PDF of the transverse distribution of autonomous vehicles under the scenario of Pattern 2, *f*_a,B_(*x*)~*B*-*Gaussian*(*μ*_1_, *μ*_2_, $\sigma_{1}^{2}$, $\sigma_{2}^{2}$) in which *μ*_1_ and *μ*_2_ are the mean of the first peak and the second peak, respectively; *σ*_1_ and *σ*_2_ are the standard deviation of the first peak and the second peak, respectively. As the bimodal Gaussian distribution proposed in this paper is symmetric with the lane centerline, *f*_a,B_(*x*) can be expressed as *f*_a,B_(*x*)~*B*-*Gaussian*(−*μ*_a,B_, *μ*_a,B_, $\sigma_{a,B}^{2}$, $\sigma_{a,B}^{2}$), in which *μ*_a,B_ was taken as 25 cm and *σ*_a,B_ was taken as 0 ~ 25 cm.

The PDF of the transverse distribution of the mixed traffic flow under Pattern 1 and Pattern 2 can be determined by replacing *F*_a_(*x*) in Equation (3) with *F*_a,U_(*x*) in Equation (9) and *F*_a,B_(*x*) in Equation (10), respectively. In addition, the fatigue life of fatigue details in OSDs under the action of vehicle loads can be evaluated as

(11) $$Y=\frac{1}{365\times N_{e}\cdot D_{e}}$$

where *N_e_* is the equivalent number of average daily truck traffic within a single lane, which was calculated by converting the actual traffic flow into the standard fatigue truck shown in Figure 4.

**Figure 12:**
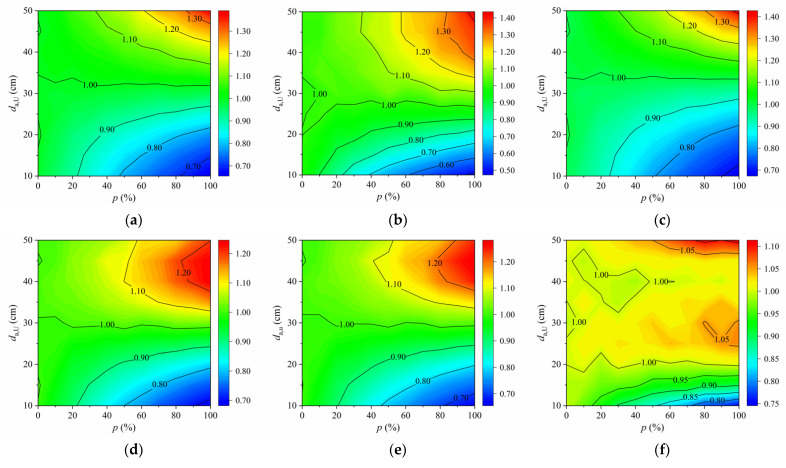

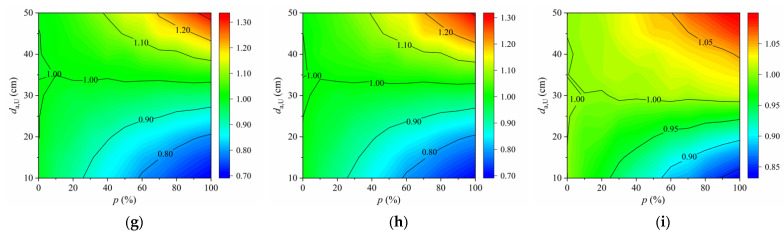
*Y*_m_/*Y*_h_ of fatigue details under the scenario of Pattern 1: (**a**) D1 in COSD; (**b**) D2 in COSD; (**c**) D3 in COSD; (**d**) D4 in COSD; (**e**) D5 in COSD; (**f**) D6 in COSD; (**g**) D4 in LWCD; (**h**) D5 in LWCD; (**i**) D6 in LWCD.

**Figure 13:**
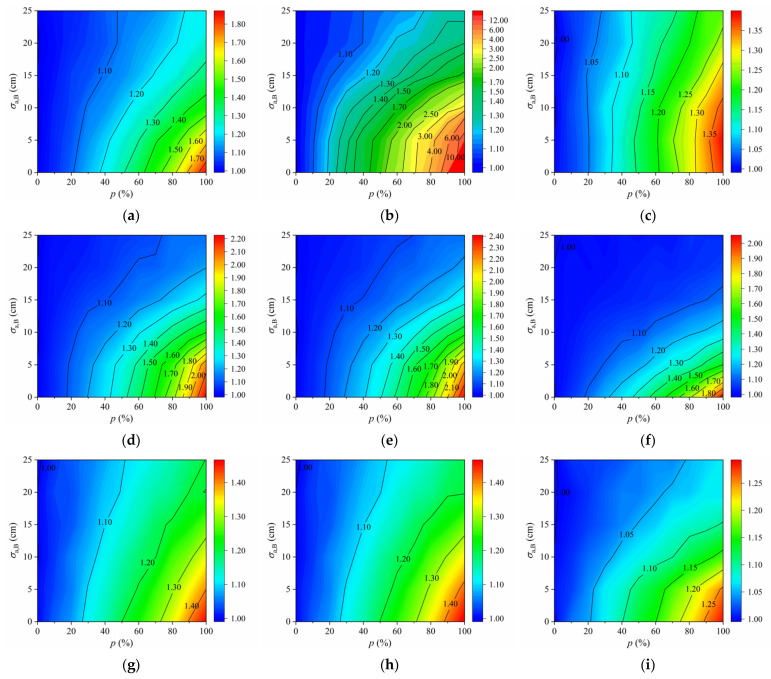
*Y*_m_/*Y*_h_ of fatigue details under the scenario of Pattern 2: (**a**) D1 in COSD; (**b**) D2 in COSD; (**c**) D3 in COSD; (**d**) D4 in COSD; (**e**) D5 in COSD; (**f**) D6 in COSD; (**g**) D4 in LWCD; (**h**) D5 in LWCD; (**i**) D6 in LWCD.

To evaluate the effectiveness of the proposed transverse distribution patterns of autonomous vehicles for extending the fatigue life of OSDs, the fatigue life of each fatigue detail under the action of the mixed traffic flow (donated as *Y*_m_) was compared with that obtained under the action of human-driven vehicles (donated as *Y*_h_) according to Equation (11). For comparison, the total number of vehicles, *N_e_*, under different scenarios was assumed to be the same. The ratios *Y*_m_/*Y*_h_ of each fatigue detail under the scenario of Pattern 1 and Pattern 2 are plotted in Figure 12 and Figure 13, respectively.

It can be found from Figure 12 that under the scenario of Pattern 1, the ratio *Y*_m_/*Y*_h_ of fatigue details in OSDs is mostly less than 1.00 and decreases with the increase in *p* when *d*_a,U_ < 30 cm. Specifically, the ratio *Y*_m_/*Y*_h_ of the fatigue detail D2 in COSD is even less than 0.50 in the most unfavorable case. Conversely, the ratio *Y*_m_/*Y*_h_ of all fatigue details is greater than 1.00 and increases with the increase in *p* when *d*_a,U_ > 35 cm. Specifically, the ratio *Y*_m_/*Y*_h_ of the fatigue detail D3 in COSD can reach 1.42 in the most favorable case. This proves the effectiveness of Pattern 1 in extending the fatigue life of OSDs. However, the ratio *Y*_m_/*Y*_h_ of most fatigue details is significantly greater than 1.00 only when *p* > 60%. Furthermore, the ratio *Y*_m_/*Y*_h_ of most fatigue details increases with the increase in *d*_a,U_, except the fatigue details D4 and D5 in COSD, for which the optimal value of *d*_a,U_ is about 45 cm. Comparing the results of fatigue details in the COSD and the LWCD, it can be found that the ratios *Y*_m_/*Y*_h_ of the same fatigue detail in these two OSDs have little difference under the same scenario.

As can be seen from Figure 13, the ratio *Y*_m_/*Y*_h_ of fatigue details in OSDs is almost larger than 1.00 under the scenario of Pattern 2. In particular, the ratio *Y*_m_/*Y*_h_ would be significantly greater than 1.00 once *p* exceeds 30% in most cases, indicating that the fatigue life of OSDs can be extended under this scenario. It can also be found that the ratio *Y*_m_/*Y*_h_ of these fatigue details increases with the decrease in *σ*_a,B_ or the increase in *p*, while the increment of the ratio for each fatigue detail is quite different in the same case. Specifically, the ratio *Y*_m_/*Y*_h_ of most fatigue details in the COSD is larger than 1.86 in the most favorable case. The maximum ratio of the fatigue detail D2 in COSD can even be larger than 12.00. However, the ratio *Y*_m_/*Y*_h_ of fatigue details in the LWCD is significantly smaller than that of the COSD under the same scenario. Compared with Pattern 1, Pattern 2 seems to be more effective in extending the fatigue life of OSDs in most cases. Nevertheless, it is necessary to avoid the negative effect of overly concentrated autonomous vehicles on the deterioration of pavement since a smaller *σ*_a,B_ results in a more positive effect of autonomous vehicles on the fatigue life of OSDs.

## 6. Conclusions

In this paper, the effects of autonomous vehicles on the fatigue life of the COSD and the LWCD were investigated, in which different transverse distribution patterns of autonomous vehicles and their proportions in the mixed traffic flow were considered. Furthermore, two distribution patterns were proposed to evaluate the feasibility of extending the fatigue life of OSDs by optimizing the transverse distribution of autonomous vehicles. Based on the results obtained from the study, the main conclusions are summarized as follows:

1. The transverse distribution of vehicles in lanes can be significantly affected by autonomous vehicles. Autonomous vehicles would be overly concentrated on the lane centerline and be more likely to pass the bridge from a more unfavorable transverse position if their transverse distribution is unconstrained, which may accelerate the fatigue damage of fatigue details and shorten the fatigue life of OSDs, especially when the proportion of autonomous vehicles in the mixed traffic flow exceeds 30%. Specifically, the fatigue damage of most fatigue details in OSDs may increase by 51% to 210% in the most unfavorable case in which all autonomous vehicles concentrate on the most unfavorable transverse position.
2. It is feasible to prevent the negative effect of autonomous vehicles on the fatigue damage of fatigue details and even extend the fatigue life of OSDs by optimizing the transverse distribution of autonomous vehicles. Compared to the uniform distribution, the bimodal Gaussian distribution is a more efficient transverse distribution pattern for autonomous vehicles, under which the fatigue life of OSDs can be extended significantly once the proportion of autonomous vehicles exceeds 30% and the fatigue life of most fatigue details in the COSD could be extended by more than 86% in the most favorable case.
3. The fatigue life of both the COSD and the LWCD can be significantly affected by autonomous vehicles. However, the fatigue performance of the LWCD is better than that of the COSD. Moreover, both the negative and positive effects of autonomous vehicles on the fatigue life of the COSD are more significant than those of the LWCD in most cases.

It should be noted that the effect of autonomous vehicles on the fatigue life of OSDs was evaluated from the perspective of finite fatigue life in this paper. It would be another interesting topic to investigate whether the risk of fatigue cracking in OSDs can be eliminated by optimizing the transverse distribution of autonomous vehicles. In addition, many factors in the real environment were not considered in this paper, including the true conditions of the traffic flow, the pavement degradation, and the robustness of the control system, which need to be evaluated in the future.

## Figures and Tables

**Table 1:** Key parameters of the FEM.

Component	Element Type	Young’s Modulus (GPa)	Poisson’s Ratio	Density (kg/m^3^)
steel plates	SHELL181	210	0.3	7850
UHPC	SOLID185	42.6	0.2	2700
short studs	BEAM189	210	0.3	7850

**Table 2:** Detailed information of fatigue details.

Fatigue Detail	Stress Type	Type of Hot Spot	Extrapolation Path	Stress Type *^1^	Detail Category
D1	Hot spot	a	Linear	*S*_X_	90
D2	Hot spot	a	Linear	*S*_Y’_/*S*_Y’’_	90
D3	Nominal	—	—	*S*_Z_	71
D4	Hot spot	a	Linear	*S*_Z_	90
D5	Hot spot	a	Linear	*S*_Y’_/*S*_Y’’_	90
D6	Hot spot	b	Quadratic	*S*_1_ *^2^	90

*^1^ The coordinate system of the stress type is illustrated in Figure 3. *^2^ *S*_1_ represents the principal stress.

## Data Availability

Not applicable.

## Notation:

COSD	conventional OSD
*D*	cumulative fatigue damage of fatigue details
DOF	degrees of freedom
*D_e_*	equivalent fatigue damage of each fatigue detail
*d*_a,U_	the maximum offset of autonomous vehicle from lane centerline
FEM	finite element model
FLM	fatigue load model
*F*_a_(*x*)	modified PDF of the transverse distribution of autonomous vehicles
*F*_a,B_(*x*)	modified PDF of the transverse distribution of autonomous vehicles when following a bimodal Gaussian distribution
*F*_a,U_(*x*)	PDF of the transverse distribution of autonomous vehicles when following a uniform distribution
*F*_h_(*x*)	modified PDF of the transverse distribution of human-driven vehicles
*F*_m_(*x*)	modified PDF of the transverse distribution of mixed traffic flow
*f*_a_(*x*)	original PDF of the transverse distribution of autonomous vehicles
*f*_a,B_(*x*)	original PDF of the transverse distribution of autonomous vehicles when following a bimodal Gaussian distribution
*f*_h_(*x*)	original PDF of the transverse distribution of human-driven vehicles
*K_C_*	fatigue strength constant corresponding to high-stress range
*K_D_*	fatigue strength constant corresponding to low-stress range
LWCD	lightweight composite OSD
*N_e_*	equivalent number of average daily truck traffic within a single lane
*N_i_*	number of stress cycles corresponding to the stress range of Δ*S_i_* at which fatigue failure occurs
*N_j_*	number of stress cycles corresponding to the stress range of Δ*S_j_* at which fatigue failure occurs
*n_i_*	number of actual stress cycles experienced by fatigue detail corresponding to stress range of Δ*S_i_*
*n_j_*	number of actual stress cycles experienced by fatigue detail corresponding to stress range of Δ*S_j_*
OSD	orthotropic steel deck
PDF	probability density function
*p*	proportion of autonomous vehicles in mixed traffic flow
RC	rib-to-crossbeam
RD	rib-to-deck
*S*_hot_	hot spot stress
*S*_0.5*t*_	stress at reference node that is 0.5 × *t* away from the node of interest
*S*_1.5*t*_	stress at reference node that is 1.5 × *t* away from the node of interest
*S*_4mm_	stress at reference node that is 4 mm away from the node of interest
*S*_8mm_	stress at reference node that is 8 mm away from the node of interest
*S*_12mm_	stress at reference node that is 12 mm away from the node of interest
*t*	thickness of plate on which the weld toe is located
UHPC	ultrahigh performance concrete
*x*	transverse offset of the vehicle from lane centerline
*Y*	fatigue life of fatigue details
*Y*_h_	fatigue life of fatigue details under the action of human-driven vehicles
*Y*_m_	fatigue life of fatigue details under the action of mixed traffic flow
Δ*σ*_C_	detail category of fatigue details
Δ*σ*_D_	constant amplitude fatigue limit of fatigue details
Δ*σ*_L_	cut-off limit of fatigue details
Δ*S_i_*	the *i*-th high-stress range
Δ*S_j_*	the *j*-th low-stress range
*μ*_1_	mean of the first peak of *f*_a,B_(*x*)
*μ*_2_	mean of the second peak of *f*_a,B_(*x*)
*μ*_a_	mean of *f*_a_(*x*)
*μ*_a,B_	absolute value of *μ*_1_ and *μ*_2_ when the bimodal Gaussian distribution is symmetric with lane centerline
*μ*_h_	mean of *f*_h_(*x*)
*σ*_a_	standard deviation of *f*_a_(*x*)
*σ*_h_	standard deviation of *f*_h_(*x*)
